# One-Pot Solvothermal Synthesis of Bi_4_V_2_O_11_ as A New Solar Water Oxidation Photocatalyst

**DOI:** 10.1038/srep22727

**Published:** 2016-03-07

**Authors:** Zaiyong Jiang, Yuanyuan Liu, Mengmeng Li, Tao Jing, Baibiao Huang, Xiaoyang Zhang, Xiaoyan Qin, Ying Dai

**Affiliations:** 1 State Key Laboratory of Crystal Materials, Shandong University, Jinan 250100, P. R. China; 2 School of Physics, Shandong University, Jinan 250100, P. R. China

## Abstract

Bi_4_V_2_O_11_ was prepared via a one-pot solvothermal method and characterized via XRD, Raman, XPS, Electrochemical impedance spectroscopy. The as-prepared Bi_4_V_2_O_11_ sample displays excellent photocatalytic activity towards oxygen evolution under light irradiation. The hierarchical structure is in favour of the spatial separation of photogenerated electrons and holes. Furthermore, the internal polar field also plays a role in improving the charge separation. Both of the two results are responsible for excellent activity of O_2_ evolution. The resulting hierarchical Bi_4_V_2_O_11_ sample should be very promising photocatalyst for the application of photocatalytic O_2_ evolution in the future.

Since the first report about photocatalytic water splitting via TiO_2_ electrode by Honda *et al.* in 1972^1^, how to convert solar energy into renewable clean chemical energy has become one of the most profound challenges^2^,^3^. Over the past few years, various effort has been put into the research on water splitting via semiconductor photocatalysis^4^,^5^,^6^,^7^, and some excellent results have been achieved^8^,^9^. Nevertheless, the efficiencies are still low and do not meet the requirements of practical applications^10^. As far as we know, the photocatalytic oxygen evolution is considered as the crucial step for the water splitting^11^. Therefore, the major challenge for water splitting is how to work out the problem of O_2_ evolution.

Currently, BiVO_4_ is the most widely investigated photocatalyst for the water splitting to produce O_2_, due to its advageous, such as nontoxicity, low cost and high chemical and thermal stability etc. Various methods were proposed to enhance the photocatalytic activity of BiVO_4_, including doping^12^, semiconductor recombination^13^,^14^, and cocatalyst depositing^11^, etc. Based on the above discussion, we can find that the current studies on O_2_ evolution mainly focused on the modification of BiVO_4_. However, the reports about exploration of new photocatalyst with the performance of oxygen evolution are limited. Therefore, further explorations on new photocatalysts with photocatalytic oxygen evolution activity are of great importance for both scientific research and practical applications.

Bi_4_V_2_O_11_ is one of newly exploited Bi-based semiconductor photocatalyst with the features of nontoxicity, low cost and high chemical and thermal stability. It has a layered structure that consists of [Bi_2_O_2_] slabs which are interleaved by (VO_3.5_□_0.5_)^2−^ (Fig. 1a)^15^,^16^. The unique layered structure is in favour of enhancing the separation of photo-generated electron-hole pairs^17^, which can improve the photocatalytic property. Moreover, this compound exhibits strong polar response^18^,^19^, which can form the internal polar electric field to further enhance the separation of photoinduced carriers^20^,^21^. So, Bi_4_V_2_O_11_ photocatalyst has attracted widespread attention for applications in the degradation of organic compounds under solar light illumination^22^,^23^. For example, Liu *et al.*^15^ prepared Bi_4_V_2_O_11_ hierarchical hollow microspheres by a facile template-free solvothermal route and exhibited excellent photocatalytic activity for the degradation of Rhodamine-B.

However, to the best of our knowledge, researches on the photocatalytic activity of Bi_4_V_2_O_11_ are limited to organic molecules degradation. Up to now, there is no a report whether the Bi_4_V_2_O_11_ photocatalyst can split water to generate oxygen. Through the preliminary observation, we could find that the Bi_4_V_2_O_11_ possess same element compositions with the BiVO_4_, which suggests their properties to be possibly similar. In order to further confirm the possibility, we have calculated density of states of Bi_4_V_2_O_11_. As shown in Fig. 1b, the VBM of Bi_4_V_2_O_11_ are mainly contributed by O 2p states, and slightly contributed by Bi 6s states, moreover, the CBM are mainly composed of V 3d states and Bi 6p states, which is basically consistent with that of BiVO_4_ (Figure S1). Based on above discussion, we speculate that Bi_4_V_2_O_11_ might have excellent performance of O_2_ evolution like BiVO_4_. Besides that, building internal polar electric field was already believed to be a usfull way to improve photocatalytic activity. Based on above discussion, the exploration of O_2_ evolution of Bi_4_V_2_O_11_ is of great importance for both scientific research and practical applications.

In this paper, we synthesized the Bi_4_V_2_O_11_ photocatalyst via a one-pot facile solvothermal method and efficient photocatalytic oxygen evolution from water was observed over Bi_4_V_2_O_11_. First princle calcaultion suggests that both the layered structure and the spontaneous electric polarization of Bi_4_V_2_O_11_ play important roles in suppresing the recombination of photogenerated charge carriers, which enable Bi_4_V_2_O_11_ to diaplay effient photocatalytic acitvity towards exygen evolution from water.

## Results and Discussion

**Figure 1:**
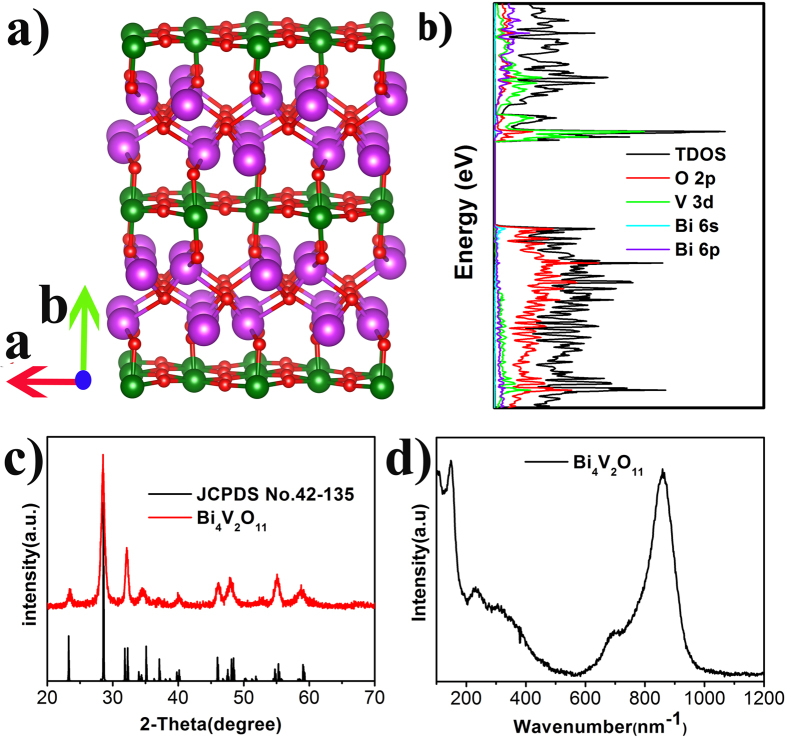
A perspective view of the Bi_4_V_2_O_11_ slab (**a**) (The big purple circles represent Bi atoms, the small red circles represent O atoms and the green circles represent V atoms, respectively.), Density of states of Bi_4_V_2_O_11_ (**b**) XRD patterns of Bi_4_V_2_O_11_ (**c**) and Raman spectra for Bi_4_V_2_O_11_ (**d**).

The XRD pattern of as-prepared Bi_4_V_2_O_11_ is shown in Fig. 1c, in which all the diffraction peaks can be perfectly indexed to the standard data for orthorhombic crystal structure of Bi_4_V_2_O_11_ (JCPDS No. 42–135). No impurity peaks were observed, indicating the high purity of Bi_4_V_2_O_11_. Raman spectra were further investigated so as to obtain more structural information of Bi_4_V_2_O_11_. The result was shown in Fig. 1d. A broad peak between 600 and 950 cm^−1^ was observed, which could be attributed to stretching modes of V-O band of VO_6_ groups. Moreover, two peaks located at about 232 and 303 cm^−1^ were found, which can be regarded as lattice modes or vibration chain bending, respectively^24^. This result also suggests the successful preparation of Bi_4_V_2_O_11_, which is in good consistent with the foregoing XRD result. SEM images (Figure S2) suggest that the morphology of the as-prepared Bi_4_V_2_O_11_ is irregular nanoparticle. And EDS results (Figure S3) suggest that the atom ratio of Bi to V is 2.14, which is in accordance with the stoichiometric value, i.e. 2.

X-ray photoelectron spectra (XPS) were further investigated to identify the surface compositions and valence states of Bi_4_V_2_O_11_, which is shown in Figure S4. Two peaks at 159.20 eV and 164.48 eV are detected, which can be attributed to Bi^3+^ 4f_7/2_ and Bi^3+^ 4f_5/2_, respectively^25^. There are three binding energy locates at 529.88, 531.25 and 532.71 eV, respectively, which are assigned to the lattice oxygen, hydroxyl groups adsorbed onto the surface of sample and oxygen vacancies. Furthermore, the binding energy peaks at 516.72 and 524.09 eV are ascribed to the V 2p_3/2_ and V 2p_1/2_, respectively, which is identical to the results of V^5+^ in Bi_4_V_2_O_11_ reported in literature^22^. The XPS results indicate that Bi_4_V_2_O_11_ with high purity was successfully synthesized.

**Figure 2:**
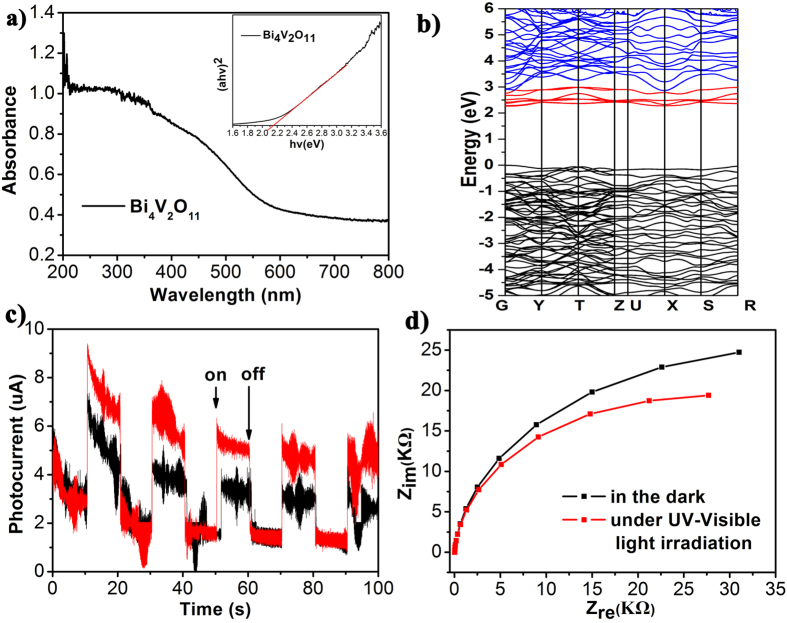
(**a**) UV-Visible DRS spectra, (inset of a) plots of (ahv)^2^ versus the photon energy (hv), (**b**) the images of energy band structure of Bi_4_V_2_O_11_, (**c**) Transient photocurrent responses of Bi_4_V_2_O_11_ under a bias of 0.4V and 0.8 V and (**d**) EIS Nyquist plots the Bi_4_V_2_O_11_ electrode in the dark and under UV-Visible light irradiation. The reference electrode was Ag/AgCl, and the electrolyte was 0.2 M Na_2_SO_4_.

Figure 2a shows the UV-Visible (UV-Vis) diffuse reflection spectra (DRS) spectra of Bi_4_V_2_O_11_. It is observed that Bi_4_V_2_O_11_ exhibits strong visible-light absorption with the absorption edge of ca. 580nm. The band gap of Bi_4_V_2_O_11_ is determined to be 2.15eV (the inset of Fig. 2a). In addition, the potentials of conduction band (CB) and valence band (VB) edges of Bi_4_V_2_O_11_ were calculated using the Mulliken electronegativity theory^26^, whose equations are listed in equation 2, 3, 4, 5. The calculated values were E_CB_ = 0.61 eV and E_CB_ = 2.76 eV, respectively. This result suggests that Bi_4_V_2_O_11_ is able to oxide water giving rise to oxygen thermodynamically. The energy band structure of Bi_4_V_2_O_11_ were also calculated and the result was shown in Fig. 2b. It is observed that both the VBM and the CBM locate at Gama point, which indicates that Bi_4_V_2_O_11_ is a direct band gap semiconductor. The calculated band gap is about 2.26 eV, which is good agreement with the experimental value.

Transient photocurrent responses were performed to investigate the photoelectric properties of Bi_4_V_2_O_11_ and the result is shown in Fig. 2c. The dark current is very low while the photocurrent increases sharply upon light illumination, and reaches a steady state quickly. As expected, the current returns quickly to its darkcurrent state when the light is turned off. This result suggests that free electron-hole pairs can be generated and efficiently separated when Bi_4_V_2_O_11_ is irradiated by UV-Vis light. The result of electrochemical impedance spectroscopy (EIS) further supports the above conclusion(Fig. 2d). The arc radius in the EIS Nyquist plot under UV-Vis light illumination is smaller than that in the dark, suggesting that the photo-generated electron-hole pairs can be well separated and efficiently transferred to the surface of Bi_4_V_2_O_11_^27^. Based on the above results, it is reasonable to conclude that Bi_4_V_2_O_11_ is a potential photocatalyst.

**Figure 3:**
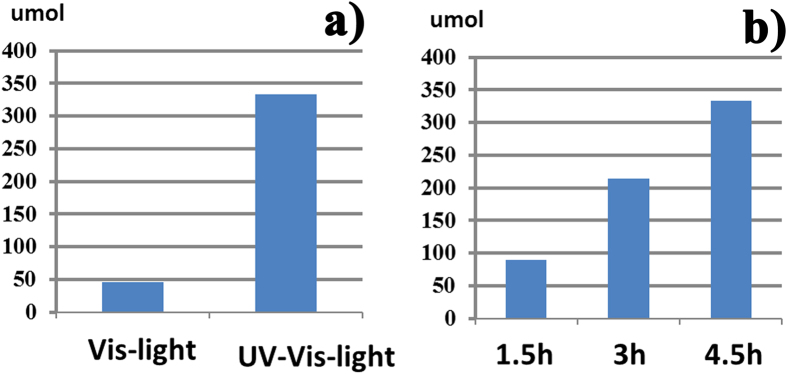
(**a**) Photocatalytic O_2_ evolution of Bi_4_V_2_O_11_ and under UV-Visible and Visible light irradiation, (**b**) the detailed image of O_2_ evolution of Bi_4_V_2_O_11_ upon UV-Visible light irradiation.

To check above assumption, photocatalytic oxygen evolution reaction was carried out over Bi_4_V_2_O_11_ in 100 mL of aqueous solution containing AgNO_3_ as a sacrificial reagent. As shown in Fig. 3a, Bi_4_V_2_O_11_ exhibits an excellent O_2_ evolution activity under both UV-Vis and Vis light irradiation. Figure 3b shows the photocatalytic O_2_ evolution, as a function of reaction time under UV-Vis light irradiation. Obviously, the amount of O_2_ evolution increases with increasing the reaction time.

The electric dipole moments were calculated in order to check whether the internal polar electric field plays an important role in the excellent photocatalytic activity of Bi_4_V_2_O_11_. The bond valences of [BiO_4_] and [VO_6_] unit were calculated using the following equation:


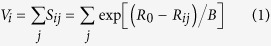


In equation, R_0_ represents the average bond length (Bi-O or V-O) and B = 0.37 is a constant. R_ij_ is the actual bond length between i and j. Moreover, S_ij_ is defined as the valence of the bond i–j. And, V_i_ is the bond valence sum of the cation i. Through the Debye equation μ = neR, the net dipole moments of [BiO_4_] and [VO_6_] units were calculated and given in Table 1. Here, n represents the total number of electrons, *e* is th*e* electron charge, *R* is the difference between the “centroids” of positive and negative charge and μ is the net dipole moment in Debye. As shown in table 1, it is observed that the dipole moments in *ab* plane offset mutually. Therefore, the direction of net dipole moment of the Bi_4_V_2_O_11_ in a unit cell is along the *c* axis (0, 0, 1) and the value is determined to be 91.032 D by calculation. From the DOS image (Fig. 1b), it is observed that V atoms makes the mainly contribution to the conduction band. So, the photo-generated electrons will transfer along V-O-V layer direction. While, the holes can transfer along Bi-O-Bi layer direction due to their significant contribution to the valence band. It is noteworthy to point out that the separated direction of photo-generated electron and hole pairs is in accordance with that of internal polar electric field. Therefore, the internal polar field would facilitate the transfer of photogenerated electron and hole pairs along opposite directions, which is advantageous of the separation of photogenerated charge carriers and consequently in favour of high photocatalytic O_2_ evolution. Time-resolved PL spectrum of Bi_4_V_2_O_11_ (Figure S5) indicates that the excited state of Bi_4_V_2_O_11_ displays a two exponential decay. i.e. 0.205 ns (82.46%), and 1.085 ns (17.54%), respectively. The longer lifetime (1.085 ns) is mostly likely due to the effect of the internal polar field.

**Figure 4:**
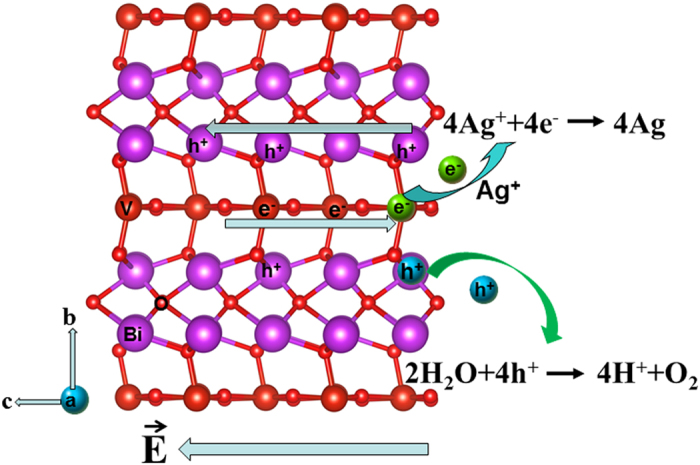
Schematic illustration of the photocatalytic process of Bi_4_V_2_O_11_.

Based on both the theoretical and experimental results, a possible photocatalytic mechanism is proposed to explain the process of photocatlytic O_2_ evolution over Bi_4_V_2_O_11_, which is shown in Fig. 4. Under light irradiation, Bi_4_V_2_O_11_ semiconductor was excited. The photo-generated electrons and holes gather in the (VO_3.5_□_0.5_)^2−^ layers and (Bi_2_O_2_)^2+^ layers, respectively, resulting in the photo-generated electrons and holes spatially separated. Moreover, the internal polar field accelerates the separation process. The photo-generated electrons on (VO_3.5_□_0.5_)^2−^ layers can be captured by Ag^+^ in the AgNO_3_ aqueous solution, generating metallic Ag. At the same time, the photo-generated holes on (Bi_2_O_2_)^2+^ layers could directly oxides water to produce O_2_. In our opinion, both Bi_4_V_2_O_11_ and BiVO_4_ are bismuth vanadium based semiconductors, and both of them are stable during the photocatalytic process. Moreover, Bi_4_V_2_O_11_ displays two advantages compared with BiVO_4_. First, the band gap of Bi_4_V_2_O_11_ (2.15 eV) is narrower than that of BiVO_4_ (2.4 eV), which means that Bi_4_V_2_O_11_ can absorb more solar light. Second, the internal polar field of Bi_4_V_2_O_11_ can facilitate the effective separation of photo-generated electron and hole pairs, which is in favour of high photocatalytic O_2_ evolution.

In summary, the hierarchical Bi_4_V_2_O_11_ photocatalyst was synthesized via one pot solvothermal method, which is verified to display excellent photocatalytic O_2_ evolution activity under light irradiation. The layered structure can effectively facilitate the spatial separation of photoinduced charges. Moreover, the intrinsic internal polar field makes the photogenerated electrons and holes move along opposite directions. The two factors are believed to be advantageous for the separation of photogenerated charge carriers and therefore responsible for excellent photocatalytic performance of Bi_4_V_2_O_11_ towards O_2_ evolution.

## Methods

### Hydrothermal synthesis of Bi_4_V_2_O_11_

All reagents used in this study were purchaseed from the Sinopharm Chemical Reagent Corporation (Shanghai, China) and they were of analytical grade without further purification. Bi_4_V_2_O_11_ photocatalyst was prepared via one-pot facile solvothermal method according to ref. 15. In a typical process, 5 mmol Bi(NO_3_)_3_·5H_2_O and 1.0 g urea were dissolved into 70 mL ethylene glycol (EG) solution with vigorous stirring,. Afterwards, 2.5 mmol NH_4_VO_3_ were added into the above solution. The pH was adjusted to 7.5 by the addition of diluent ammonia water. The resulting precursor suspension was transferred into a 100 mL Teflon-lined stainless autoclave and maintained at 453 K for 24 h. And then, the reactor was allowed to cool to room temperature naturally. The resulting product Bi_4_V_2_O_11_ was washed with deionized water and absolute ethanol for several times and dried at 333 K for 6 h in oven.

### Characterization

XRD pattern of the Bi_4_V_2_O_11_ was recorded on a Bruker AXS D8 advance powder diffractometer with Cu KαX-ray radiation. The morphology was investigated by a scanning electron microscopy (SEM, Hitachi S-4800), and the diffuse reflection spectra (DRS) by a Shimadzu UV 2550 recording spectrophotometer. XPS measurement was carried out on a VG MicroTech ESCA 3000 X-ray photoelectron spectroscope with a monochromatic Al-Ka source to explore the elements on the surface. The time-resolved fluorescence spectrum was measured via Edinburgh FLS920 PL. Steady state fluoresce was performed via using an Edinburgh FS920 high sensitivity fluorescence spectrometer.

### Computation

The spin-polarized density function theory (DFT) calculations were performed by Vienna ab-inito simulation package (VASP). The generalized gradient approximation (GGA) of Perdew, Burke, and Ernzerhof (PBE) was employed for the exchange-correlation functional. The cut-off energy of 400 eV was adopted for the plane-wave basis set. The convergence threshold for self-consistent iteration is set at 10-6 eV, and all atomic structures were fully relaxed until the residual forces on all atoms were smaller than 0.01 eV/Å.

According to the Mulliken electronegativity theory, the potentials of the conduction band (CB) and valence band (VB) edges of Bi_4_V_2_O_11_ were calculated, whose equations is following:


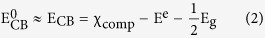



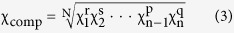



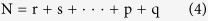



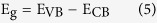


**χ**_**comp**_ represents the absolute electronegativity of the semiconductors depending on the species and number of the constituent atoms and given by geometric mean of the absolute electronegativity of each atom and the total number of atoms (equations (3) and (4); E^e^ is defined as the energy of free electrons on the hydrogen scale (Ee = ~4.5 eV), in addition, Eg is the band gap energy of the semiconductor. According to the above equation (3) and (4), **χ**_**comp**_ value is evaluated to be 6.30 eV. And then, the values of E_CB_ and E_VB_ are calculated according to the equation (2) and (5), respectively.

### Photocatalytic water oxidation activity

The photocatalytic O_2_ evolution reaction was performed in a quartz reactor at constant temperature with circulation of water through the internal U-type glass tube from a thermostatic bath. The typical experimental procedure is as follows: first, 100 mg samples were dispersed in 100 mL of aqueous AgNO_3_ solution (0.015 M) and sealed the reactor. Subsequently, argon flow has been purged the solution for 30 min to drive away the residual oxygen. And then, the mixture solution was irradiated on the 300 W Xe lamp (PLS-SXE300, Beijing Trusttech Co., Ltd.). The evolution amount of O_2_ gas was measured with the gas chromatography with a thermal conductivity detector.

### Electrochemical performances

Photocurrent measurements for Bi_4_V_2_O_11_ were carried out with a CHI 660 C electrochemical workstation. A 300 W Xe arc lamp was utilized as the light source. The Bi_4_V_2_O_11_ sample (10 mg) was spin coated on a 1.5 × 1.5 cm^2^ ITO glass electrode. The ITO glass was used as working electrodes. In addition, a Pt was employed as the counter electrode and a saturated calomel electrode was used as reference electrode. 0.2 M Na_2_SO_4_ solution was used as the electrolyte. The electrochemical impedance spectroscopy (EIS) was performed at the open circuit potential.

## Additional Information

**How to cite this article**: Jiang, Z. *et al.* One-Pot Solvothermal Synthesis of Bi_4_V_2_O_11_ as A New Solar Water Oxidation Photocatalyst. *Sci. Rep.* **6**, 22727; doi: 10.1038/srep22727 (2016).

## Supplementary Material

Supplementary Information

## Figures and Tables

**Table 1:** The total value of dipole moments of VO_6_ and BiO_4_ groups in unit cell of Bi_4_V_2_O_11._

	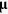 _x_	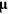 _y_	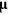 _z_
[VO_6_]	0	0	−7.102
[BiO_4_]-1	4.930	16.234	14.930
[BiO_4_]-2	4.930	−16.234	14.930
[BiO_4_]-3	−4.930	16.234	14.9303
[BiO_4_]-4	−4.930	−16.234	14.9303
Net dipole moments	0	0	91.032
