# Supplementary material for: One-Pot Solvothermal Synthesis of Bi4V2O11 as A New Solar Water Oxidation Photocatalyst
Source: Sci Rep. 2016 Mar 7;6:22727. doi: 10.1038/srep22727 (PMC4779990; doi:10.1038/srep22727)
Supplement: Supplementary Information [file srep22727-s1.pdf]

Supplementary Information

for

# One-Pot Solvothermal Synthesis of $Bi_4V_2O_{11}$ as A New Solar Water Oxidation Photocatalyst

Zaiyong Jiang[1], Yuanyuan Liu[1,]*, Mengmeng Li[2], Tao Jing[2], Baibiao Huang[1,]*, Xiaoyang Zhang[1], Xiaoyan Qin[1] and Ying Dai[2]

[1] State Key Laboratory of Crystal Materials, Shandong University, Jinan 250100 (P. R. China)

[2] School of Physics, Shandong University, Jinan 250100 (P. R. China)

Corresponding Author:

Yuanyuan Liu (Y.Y.L.), yyliu@sdu.edu.cn

Baibiao Huang (B. B. H.), bbhuang@sdu.edu.cn

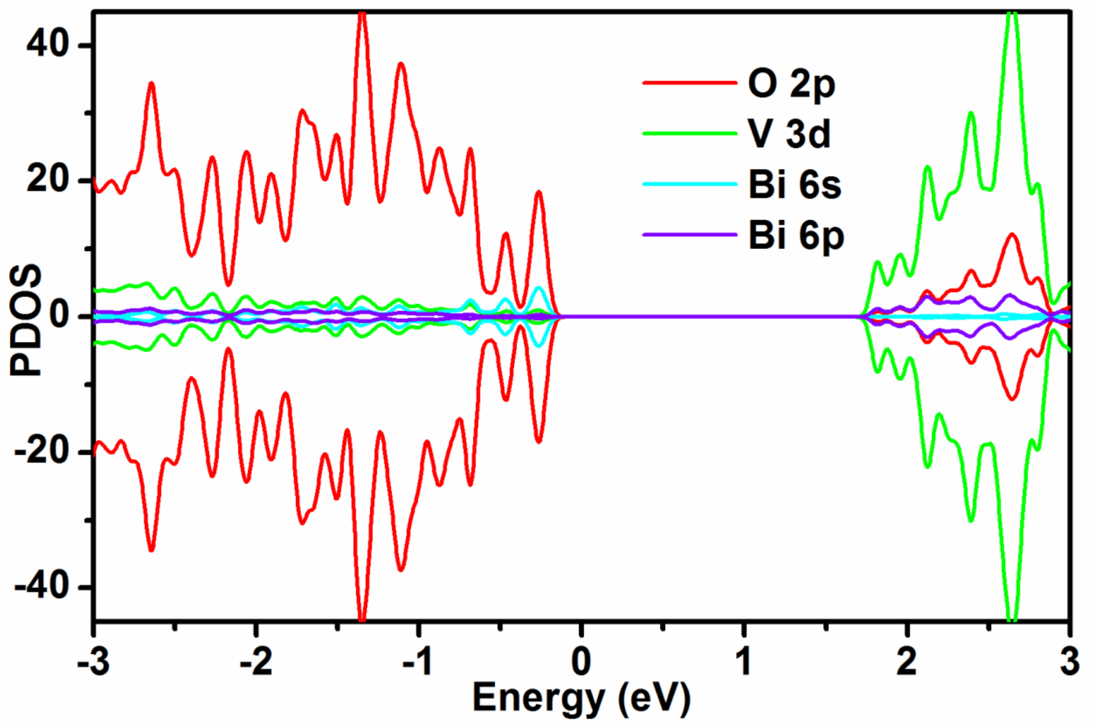


**Figure S1.** Density of states of $BiVO_4$

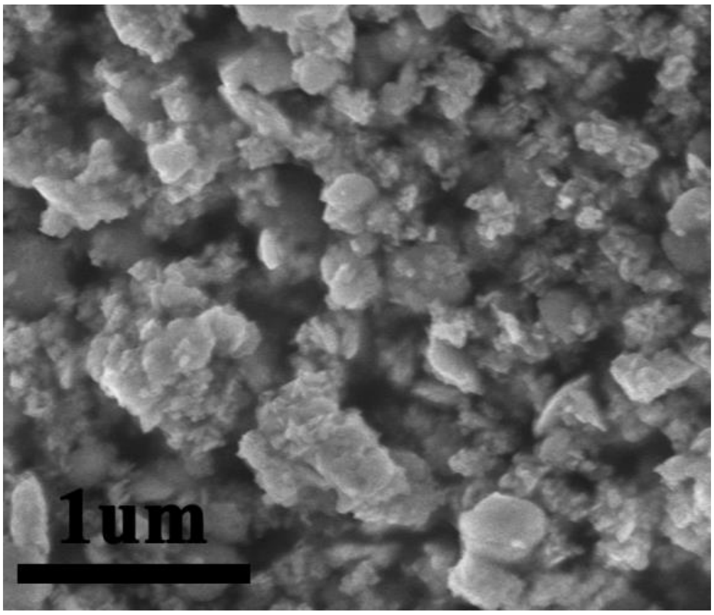


**Figure S2.** SEM image of $Bi_4V_2O_{11}$

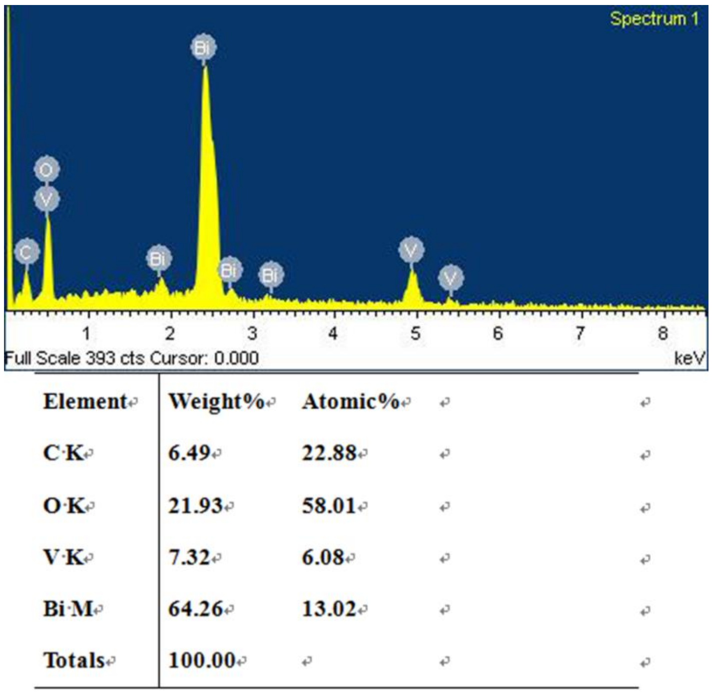


| Element | Weight% | Atomic% |
|---|---|---|
| C K | 6.49 | 22.88 |
| O K | 21.93 | 58.01 |
| V K | 7.32 | 6.08 |
| Bi M | 64.26 | 13.02 |
| Totals | 100.00 | |

**Figure S3.** EDX spectrum and detailed information about the composition of $Bi_4V_2O_{11}$ sample

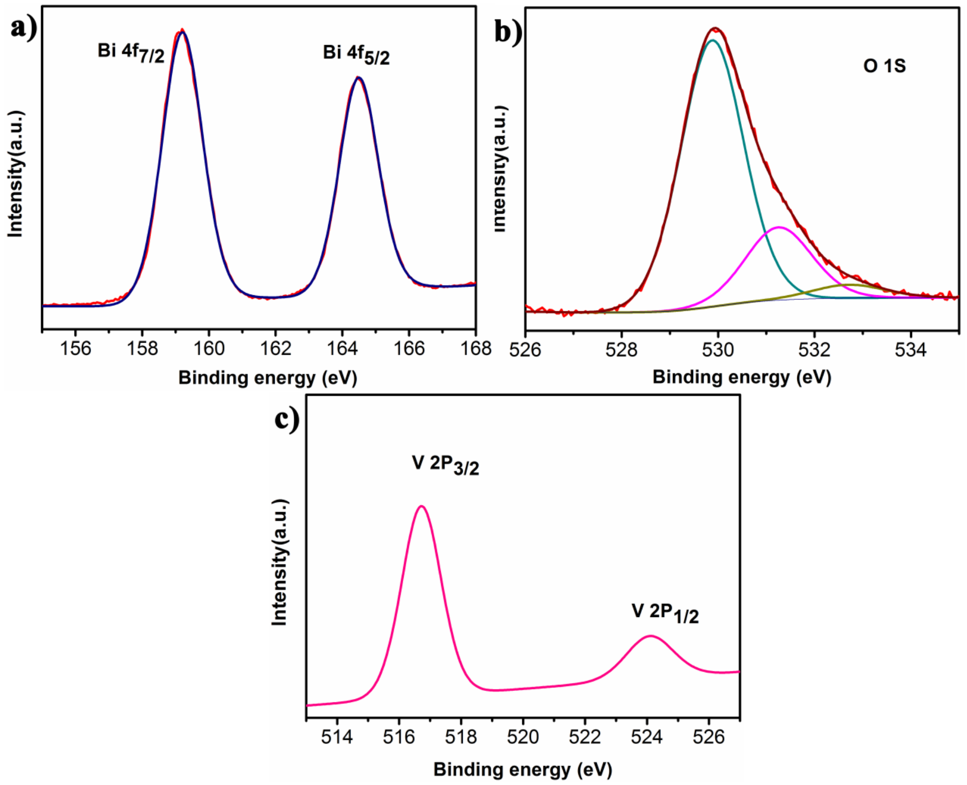


**Figure S4.** XPS spectra of the $Bi_4V_2O_{11}$ sample: (a) the Bi 4f, (b) the O 1s and (c) the V 2p

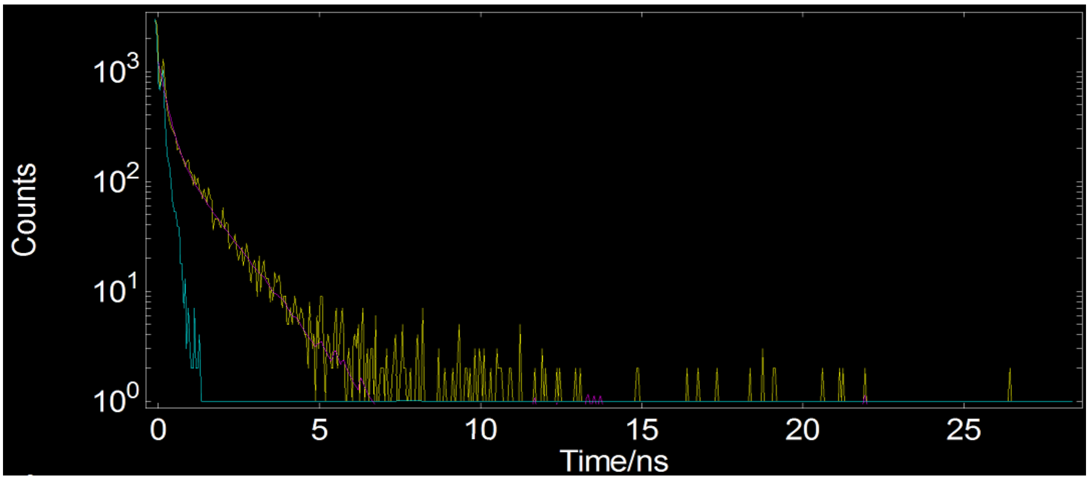


**Figure S5.** Time-resolved PL spectrum for $Bi_4V_2O_{11}$ detected at 430 nm. The excitation source is a 380 nm laser.

The detection wavelength is determined according to the steady state PL spectrum (Figure S6), which displays a strong emission centered at 430nm.

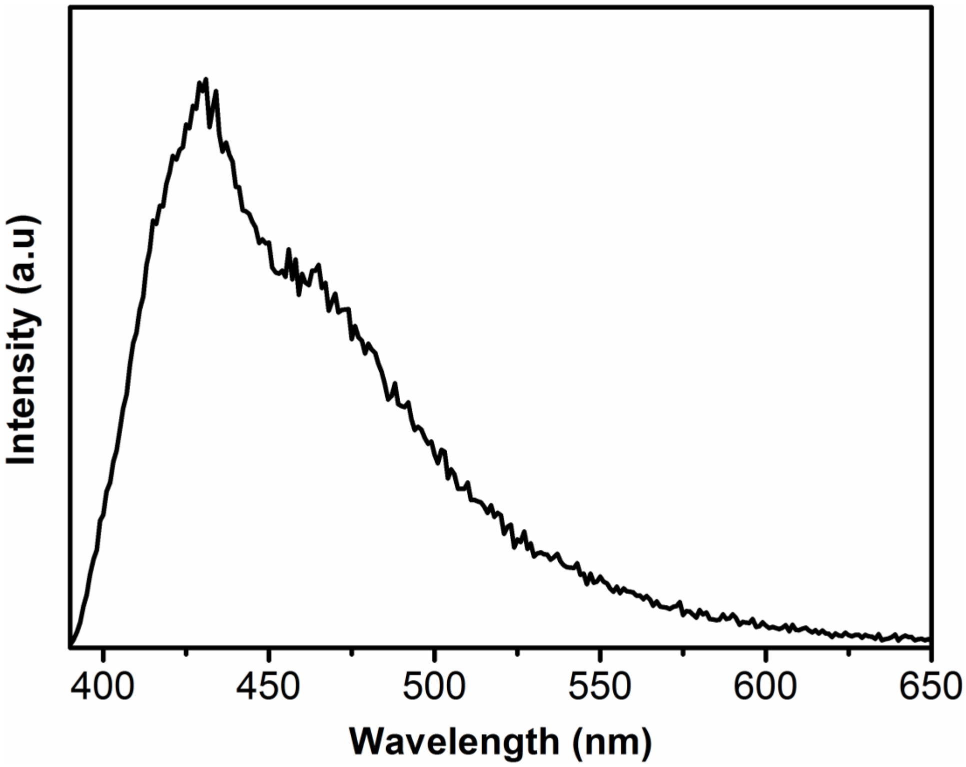


**Figure S6.** Steady state PL spectrum of $Bi_4V_2O_{11}$, and the excitation wavelength is 350 nm
